# Efficient Delivery of Triptolide Plus a miR-30-5p Inhibitor Through the Use of Near Infrared Laser Responsive or CADY Modified MSNs for Efficacy in Rheumatoid Arthritis Therapeutics

**DOI:** 10.3389/fbioe.2020.00170

**Published:** 2020-03-17

**Authors:** Xiaonan Zhang, Xin Zhang, Xipeng Wang, Tao Wang, Bin Bai, Na Zhang, Yanjiao Zhao, Yang Yu, Bing Wang

**Affiliations:** ^1^Institute of Biochemistry and Molecular Biology, College of Life and Health Sciences, Northeastern University, Shenyang, China; ^2^Department of Rheumatology and Immunology, China-Japan Union Hospital of Jilin University, Changchun, China

**Keywords:** rheumatoid arthritis, triptolide, miR-30-5p, NIR laser-responsive, mesoporous silica nanoparticles

## Abstract

Rheumatoid arthritis (RA) is a chronic autoimmune inflammatory disease for which treatment focuses on suppressing an overactive immune system and maintaining the physiological balance of synovial fibroblasts (SFs). We found that miR-30-5p was highly expressed in rheumatoid arthritis synovial fibroblasts (RASFs). Subsequently, we predicted that phosphatidylinositol 3-kinase regulatory subunit 2 (PIK3R2) might be a putative target of miR-30-5p. Recent studies have reported that PIK3R2 can maintain the physiological homeostasis of RASFs. Therefore, miR-30-5p inhibitor has the potential to be used in the treatment of RA, but low levels of miR-30-5p inhibitor internalization affect its application. Triptolide (TP) is an effective drug in the treatment of RA but induces severe toxicity and has a narrow therapeutic window. In this study, the cell internalization performance of miR-30-5p inhibitor was improved by loading it into cell membrane penetrating peptide (CADY)-modified mesoporous silica nanoparticles (MSNs), and the toxicity of TP was decreased by loading it into a controlled drug release system based on MSNs. The nanodrug carrier was constructed by filling a phase-change material (PCM) of 1-tetradecanol and drugs into MSNs that could be triggered by an NIR laser with thermo-chemo combination RA therapy. Our results show that the miR-30-5p inhibitor-loaded MSNs@CADY significantly inhibited RASF proliferation and increased apoptosis. In addition, MSNs@PCM@TP under 808 nm laser irradiation were effective in downregulating immune system activation in an RA rat model. Finally, the results of a pharmacodynamics study showed that the combination of MSNs@CADY@miR-30-5p inhibitor and MSNs@PCM@TP under 808 nm laser significantly increased the effectiveness of RA treatment. These findings provide a novel understanding of RA pathogenesis and a theoretical basis for RA treatment.

## Introduction

Rheumatoid arthritis (RA) is a complex autoimmune disorder with two main characteristics: hyperactivity of the immune system and abnormal proliferation of synovial fibroblasts (SFs) (Zwerina et al., 2005; Onozaki, 2009; Mullen and Saag, 2015; Collison, 2016). Current treatment for RA mainly focuses on maintaining the physiological homeostasis of synovial cells and suppressing the activity of the immune system. It is believed that damage to articular cartilage and bone tissue in RA is mainly caused by the activation and proliferation of synovial cells. The activation and proliferation of synoviocytes are closely related to apoptosis. The PI3K/AKT signaling pathway activity is prominent in SFs, and its abnormal activation can create an imbalance between SF proliferation and apoptosis (Qu et al., 2019).

PIK3R2 is a negative regulator of the PI3K/AKT signaling pathway. In 2016, Zhao et al. reported that miR-126 targeting of PIK3R2 promotes the proliferation of rheumatoid arthritis synovial fibroblasts (RASFs) and resistance to apoptosis by regulating PI3K/AKT (Gao et al., 2016). Downregulation of miR-126 may indirectly inhibit the PI3K/AKT signaling pathway by targeting PIK3R2, disrupting the balance between RASF survival and death. It has been suggested that PIK3R2, which maintains the physiological homeostasis of SFs (Gao et al., 2016), may be a potential target for the treatment of RA.

Many Chinese herbal medicines, such as Tripterygium Wilfordii Hook F (TWHF), which is the most prominent, have been reported to be effective in treating RA (Fan et al., 2018). Triptolide (TP), an epoxy diterpenoid lactone compound, is considered an important anti-inflammatory and immune inhibitory component of TWHF. The anti-arthritis activity of TP has also been reported (Kong et al., 2013; Li et al., 2014; Fan et al., 2016). Many clinical trials have also confirmed that TP has a good anti-inflammatory effect, especially in RA. Unfortunately, the hepatotoxic, nephrotoxic, and hematological toxic effects of TP limit its clinical applicability (Xue et al., 2011; Zhang et al., 2012).

Mesoporous silica nanoparticles (MSNs) have been widely used as pharmaceutical carriers, and the surface of MSNs can be easily modified by polypeptides or other functional groups. For example, cell penetrating peptides such as CADY have been widely used to modify MSNs to facilitate the effective entry of drug carriers into cells (Cai et al., 2017; Dowaidar et al., 2017). The porous structure of MSNs enhances their release performance and their drug load capacity, the effect of which prevents the side effects caused by locally high drug concentrations administered in a short time, which can be problematic when drugs are administered alone (Jeong et al., 2015).

Drug release can be controlled by using photothermal materials, such as near infrared response photothermal controlled-release drug carriers by irradiating the material with near infrared laser, and the side effects of the loaded drug can be reduced. Indocyanine green (ICG) is a drug approved by Food and Drug Administration (FDA) and widely used in clinical liver and kidney function evaluation with relatively high safety (Saxena et al., 2004; Manchanda et al., 2010). ICG has been used as a photothermal material in RA treatment (Tang et al., 2017). Phase-change materials (PCMs) are a kind of material with great latent heat hat and can change between solid and liquid at a relatively constant temperature (Choi et al., 2010). In this study, 1-tetradecanol was selected as the photothermal response phase change material. The melting point of tetradecanol is 38–39°C (Moon et al., 2011). Moreover, the material has good biocompatibility and low oral toxicity.

The drug TP was blended with ICG, which exerts photothermal effects into tetradecanol, and the tetradecanol blends were loaded into the channels and internal cavities of MSNs at a temperature higher than that of tetradecanol to form a laser responsive drug controlled-release system. Because tetradecanol (hereinafter referred to as PCM) is solid at normal temperature, the adriamycin and ICG loaded into the system are “solidified” in tetradecanol, and thus, the controlled-release system is characterized by of “zero drug release” during the delivery process. When the system is irradiated by near-infrared laser (808 nm), the indocyanine green in the system will produce heat and melt the tetradecanol, thus releasing TP and finally fulfilling the purpose of photothermal controlled-release of drugs.

Here, miR-30-5p was identified to be overexpressed in 80% (32 of 40) of the clinical RA tissues examined and verified that the miR-30-5p inhibitor was a potent reagent that relieved RA through PIK3R2 pathways. Furthermore, we combined a miR-30-5p inhibitor with TP, a traditional drug for treatment of RA, and delivered a miR-30-5p inhibitor with a CADY-modified MSNs to deliver TP with near infrared response photothermal controlled-release MSNs. We ultimately obtained an outstanding result: the combination of the MSNs@CADY@miR-30-5p inhibitor and MSNs@PCM@TP under 808 nm laser significantly reduced the symptoms of RA in the joints of rats. These findings provide a novel understanding of RA pathogenesis and a theoretical basis for RA treatment.

## Materials and Methods

### Ethics Statement

The patients included in the present study provided written informed consent. The study was approved by the Ethical Board of China-Japan Union Hospital of Jilin University.

### Sample Preparation and Cell Culture

Forty consecutively RA patients (28 females and 12 males; aged 29–59 years) treated at the Immuno-Rheumatology Clinic from January 1 to December 31, 2018, and 15 volunteers were joined the study. The inclusion criteria were as follows: patient participation was approved by the Hospital Human Ethics Committee, and each participant signed an informed written consent; all patients with RA fulfilled the criteria of the American College of Rheumatology (Altman et al., 1986; Arnett et al., 1988); the average time of clinical treatment was 7.45 ± 3.26 days; according to the Larsen classification system (Larsen et al., 1977), weight-bearing anteroposterior and lateral X-ray photos of the affected knee were taken, the patients we selected were all candidates for early synovectomy, and all radiographs were evaluated by the same experienced orthopedists; no contraindication to anesthesia or surgery; and no other major diseases. All volunteers were patients with joint injury who were admitted to China–Japan Union Hospital of Jilin University for joint repair, aged 34–48 years (mean age of 39.9 years). Exclusion criteria were patients who had ever been treated with disease-modifying antirheumatic drug trerapy, absence of joint swelling and limited range of motion, or above Larsen stage II in the operated knee joint.

Rheumatoid arthritis synovial fibroblasts were obtained during arthroplasty or synovectomy from forty patients with RA following the method described below. The synovial tissue specimens were washed five times with Hank’s buffer (pH 7.5) 5 min and then minced and placed in a 10 cm^2^ culture flask with 4 ml of DMEM (Gibco BR) and 120 μl of type II collagenase, and then incubated at 37°C for 6 h. Then, 3 ml of trypsin without EDTA was used to detach the cells from the flask for subsequent screening. Cultured RASFs from passages 4–10 were used for experiments in this study.

The human fibroblast-like synoviocyte cell line (HFLS) was obtained from Cell Applications, Inc. (San Diego, CA, United States). A human renal epithelial cell line (293 cells) and a human hepatocyte cell line (HL-7702 cells) were purchased from Feng Hui Biotechnology, Co., Ltd. The cells were cultured in DMEM (HyClone, Thermo Fisher) supplemented with 10% FBS (HyClone, Thermo Fisher), 1% penicillin streptomycin (1000 U/ml) (Gibco, Invitrogen). All cell lines were cultured in a cell incubator at 37°C under 5% CO_2_

### RNA Isolation and qRT-PCR

TRIzol reagent (Thermo Fisher Scientific) was used for total RNA extraction. One milliliter of Trizol was added to selected cells, and the mixture was shaken violently and then let stand for 5 min at 25°C. Next, 200 μl of chloroform was added, and the mixture was shaken violently, then place the mixture at 25°C for 5 min, after 12,000 rpm centrifuging at 4°C for 15 min. 500 mL of supernatant and 300 μl of isopropanol was gently mixed, then place the mixture for 10 min at 25°C, and centrifuged at 12,000 rpm at 4°C for 15 min, the supernatant was then discarded. Next, 1 mL 75% alcohol was added to the centrifuge tube, which was gently shaken, then place the mixture for 5 min at 25°C, and centrifuged at 7500 rpm at 4°C for 15 min, the supernatant was discarded. RNA free water (15 μL) was added to the centrifuge tube, and the precipitate was gently blown for dissolution. The reverse transcription procedure was 42°C for 2 min, 50°C for 15 min, and 85°C for 2 min. After the reaction, the cDNA was stored at −80°C. cDNA was synthesized through reverse transcription using an RT reagent kit (BEENbio, Shanghai, China). The mRNA levels of the genes were analyzed by qRT-PCR using a 2 × SYBR qPCR kit (BEENbio, Shanghai, China), as described in the manufacturer’s protocols. Data in all panels are representative of three independent experiments with six replicates per detection. The results were analyzed according to the 2^–ΔΔ*Ct*^ formula. The primers used in this paper are shown in Supplementary Table 1.

### Western Blot Analysis

Fifty micrograms of protein was resolved on 10% SDS-PAGE gels and transferred to PVDF membranes (Millipore, Billerica, MA, United States). The membranes were incubated with primary antibodies and a secondary antibody labeled with HRP. The following primary antibodies were used: rabbit anti-PIK3R2 (PA5-84807, Thermo Fisher, United States), anti-PI3K (3811S, CST, United States), anti-p-PI3K (4228S, Cell Signaling Technology, United States), anti-AKT (4691S, CST, United States), anti-p-AKT (4060S, CST, United States), anti-β-actin (4970S, CST, United States), and rabbit anti-human PTPN22 (ab182239, Abcam, United States).

### Enzyme Linked Immunosorbent Assay

After various treatments, serum was extracted from the fresh blood of the experimental and control rats. The samples were diluted in 1 × dilution buffer to be in the range of concentrations used in the assay. IL-2 enzyme linked immunosorbent assay (ELISA) was performed using an ELISA kit from R&D Systems (D2050), and IL-6 and TNF-α ELISA were performed follow the kit instruction (ab46027 and ab181421). The levels of IL-2, IL-6 and TNF-α released into the supernatant were analyzed by ELISA, as described in the manufacturer’s instructions. All ELISA measurements were performed in triplicate.

### Cell Proliferation Assay

MTT assay was used to detect the cell proliferation. Briefly, the cells were seeded in 96-well plates 5 × 10^3^ per well. All cell groups were incubated for 24, 48, or 72 h. MTT was added to each well, and the cells were sustained and incubated at 37°C for 4 h with 5% CO_2_. The solution was then discarded, 300 μl of dimethyl sulfoxide (DMSO) was added to each well, and the cells were shaken for 15 min at 25°C to dissolve the crystals. The absorbance of the samples was checked at 570 nm using an ELX800 universal microplate reader (Biotek Instruments, Inc.). Experiments were replicated three times.

### Annexin V and Propidium Iodide (PI) Staining

Apoptosis of nanoparticle-treated cells was assessed by flow cytometry by labeling the cells with FITC-Annexin V and PI assay. Cells were cultured at a density of 6 × 10^4^ per 6 cm dish and grown overnight. Then, 100 μg/ml MSNs, MSNs@CADY, MSNs@CADY@miR-30-5p or MSNs@CADY@miR-30-5p inhibitor were added to the cells, and incubated for 48 h. Washing with PBS for three times, the cells were incubated at 25°C with Annexin V-FITC and PI stain in darkness for 10 min. Samples consisting of 10,000 stained cells were analyzed using flow cytometry.

### Synthesis of 120 nm MSN Nanoparticles

The MSNs were synthesized as (Sha et al., 2018) reported with some modifications (Rosenholm et al., 2010; Wang et al., 2015). The 120–150 nm MSNs were first synthesized using aminotrimethoxysilane (APS) as the amine provider, TMOS as the silica precursor, and cetyltriethylammnonium bromide (CTAB) as the structure-directing agent (Rosenholm et al., 2009). Two hundred milligrams of CTAB (Aldrich, St. Louis, MO, United States) was dissolved in 150 ml of 2M NaOH and 48 ml of water and heated to 80°C. Next, 1.2 ml of tetraethyl orthosilicate (Aldrich, 98%) was added. After incubation for 15 min at 80°C, 300 μl of 3-(trihydroxysilyl) propyl methylphosphonate (Aldrich, 42%) was added, then stirred for 2 h. The particles were collected after 12,000 rpm centrifugation and washed with methanol three times.

### CADY Attachment

The self-assembled peptide CADY (GLWWKAWWKAWWK SLWWRKRKRKA) was purchased from Sangon Biotech Company. The CADY peptide was covalently conjugated to MSN-COOH using EDC and NHS. Two milligrams of MSN-COOH was dissolved in 1 ml of MES buffer (pH 6.0), and then, 4 mg of EDC and 4.2 mg of NHS were added. Then, the mixture was stirred for 4 h at 4°C. Subsequently, 100 μl of CADY antibody solution (10 mg/ml) was added and stirred for 4 h at 4°C. Then, it was washed three times with PBS to remove excess EDC. NHS, CADY and MSNs@CADY were suspended in 450 mM miR-30-5p inhibitor, after stirring for 48 h in the dark, MSNs@CADY@miR-30-5p inhibitor was collected by centrifugation, following PBS washing for three times, and vacuum dried overnight at room temperature.

### Cellular Uptake and Internalization

A total of 2 × 10^4^ HFLS cells were seeded in glass-bottom plates (35 mm, Corning Incorporated) in DMEM with 10% FBS and incubated at a final concentration of 50 μg/ml FITC-labeled MSNs and FITC-labeled MSNs@CADY for 24 h. The cells were incubated with 60 nM LysoTracker Red DND-99 (Beyotime Institute of Biotechnology, Haimen, China) for 1 h at 37°C. After washing with PBS, the cells were fixed with 4% paraformaldehyde and stained with 10 μg/ml DAPI (4′,6-diamidino-2-phenylindole, Sigma). The cells were washed three times with PBS and mounted. Micrographs were first observed under a Nikon fluorescence microscope (Nikon Eclipse Ti-S, CCD: Ri1) and then under a laser scanning confocal microscope (Leica TCS sp5, Germany) used for confocal luminescence imaging with a 63 × oil immersion objective lens.

### Preparation of MSNs@PCM@TP

Since the melting point of PCM tetradecanol is 38–39°C, it is necessary to sufficiently mix ICG and PCM for loading into MSNs at a temperature higher than the melting point. First, 100 mg of tetradecanol was heated to 60°C to fully melt, and then, 10 mg of ICG and 15 mg of TP were added and thoroughly mixed by magnetic stirring for 2 h (Kalaria et al., 2009). Then, 100 mg MSNs were mixed with a large amount of chloroform, added to the PCM mixture solution, and stirred for 2 h at 60°C. During this mixing process, with the volatilization of trichloromethane, the PCM@TP-ICG mixture slowly infiltrates the mesopore cavities of MSNs through the mesopores. Finally, excessive hot water is added to the above preparation system to produce two phases that are obviously incompatible with each other: the MSNs water phase with PCM@TP-ICG and the chloroform phase with PCM@TP-ICG not encapsulated. The two phases were separated rapidly, and the water phase was immersed in an ice water solution for cooling for 1 min and then centrifuged at 1,1000 rpm for 5 min. The precipitates were washed with precooled deionized water 8 times and then freeze dried for 24 h at −50°C and 0.8 mbar in vacuum. The MSNs loaded with PCM@TP-ICG were recorded as MSNs@PCM@TP. In addition, with the same preparation method, the system constructed without TP is recorded as MSNs@PCM.

### Testing of miR-30-5p and TP Loading Capacity of MSNs@CADY@miR-30-5p Inhibitor and MSNs@PCM@TP

MSNs@CADY was suspended in 450 mM miR-30-5p and miR-30-5p inhibitor. After stirring for 48 h in the dark, MSNs@CADY@miR-30-5p and MSNs@CADY@miR-30-5p inhibitor were collected by centrifugation, washed with PBS three times, and vacuum dried overnight at 25°C. The concentrations of miR-30-5p and miR-30-5p inhibitor were measured with a Nanodrop 3000 spectrophotometer (Thermo Fisher). The miR-30-5p loading capacity of the nanoparticles was calculated based on changes in miR-30-5p concentration before and after loading. The concentration of TP was measured by UV-Vis spectroscopy at 218 nm, and the TP-loading capacity of MSNs@PCM@TP was calculated according to the changes in TP concentration before and after loading by using UV-Vis spectroscopy at 218 nm.

For the miR-30-5p and TP drug release experiment, MSNs@CADY@miR-30-5p inhibitor and MSNs@PCM@TP were dispersed in 15 ml of PBS (pH 7.4) in semipermeable dialysis bags at 37°C with gentle shaking then take 4 ml of the released medium and add another 4 ml fresh medium. The amount of released TP was measured by UV-Vis spectroscopy at 218 nm. The amount of released miR-30-5p inhibitor was measured by a Nanodrop 3000 spectrophotometer.

### Animals and Experimental Procedures and RA Evaluation

A collagen-induced RA rat model was generated according to a previous report (Wang et al., 2018). Female Sprague-Dawley rats (8 weeks old) were purchased from the Animal Center of Northeastern University. All animal experimental procedures were approved by the Laboratory of Animal Ethical Committee of Northeastern University. The rats were randomly allocated into eight groups (*n* = 6), and the rat grouping administration method and doses are shown in the Table 1.

**TABLE 1:** The appropriate dose and method of administration in rats.

**Group**	**Administration method**	**Drugs**	**Dose**
Control group	I.P.	PBS (pH 7.5)	2 ml/time
RA model group	I.P.	PBS (pH 7.5)	2 ml/time
Methotrexate (MTX) group	I.P.	MTX	2.5 mg/kg
TP group	I.P.	TP	50 μg/kg
MSNs@PCM@TP group + 808 nm laser	I.P.	MSNs@PCM@TP	100 μg/ml
MSNs@PCM@TP group	I.P.	MSNs@PCM@TP	100 μg/ml
MSNs@CADY@miR-30-5p inhibitor group	I.C.	MSNs@CADY@miR-30-5p inhibitor	100 μg/ml
MSNs@CADY@miR-30-5p inhibitor + I.P. MSNs@PCM@TP + 808 nm laser group	I.C. (MSNs@CADY@miR-30-5p inhibitor), I.P. MSNs@PCM@TP + 808 nm laser	MSNs@CADY@miR-30-5p inhibitor + I.P. MSNs@PCM@TP + 808 nm laser	100 μg/ml MSNs@CADY@miR-30-5p inhibitor + 100 μg/ml I.P. MSNs@PCM@TP + 808 nm laser

In brief, except for rats in the control group, the rats in each group were intradermally injected with 600 μg of bovine type II collagen in 50% complete Freund’s adjuvant, and a booster was administered with the same dose of CII in 50% incomplete Freund’s adjuvant 14 days later. Starting 15 days after the first immunization, the rats in the respective groups were administered different nanodrug treatments. The concentrations of the different nanoparticles were 100 mg/ml, the dose of I.C. MSNs@CADY and I.C. MSNs@CADY@miR-30-5p inhibitor was 30 ml/kg, and the dose of I.P. MSNs@PCM@TP was 55 ml/kg. The rats were given administered the drug every 7 days, while the control group and the RA model group received only saline injections.

The symptoms were classified as follows: none, weak, mild, moderate, and severe with scores from 0 to 4, respectively (Le Goff et al., 2009). The highest possible cumulative score of a single rat was 16.

### Characterization

The morphology and structure of the products were characterized using a SEM (S-4800) and a TEM (JEOL JEM-2100F) at accelerating voltages of 5 kV and 200 kV, respectively. The pore-size distribution of the products was determined by dynamic light scattering (DLS) using a Malvern Zeta Seizer instrument. N_2_ adsorption-desorption isotherms were measured using a Micromeritics ASAP 2020 M porosity analyzer. The samples were outgassed at 573 K for at least 2 h in a vacuum. The Brunauer-Emmett-Teller (BET) and Barrett-Joyner-Halenda (BJH) methods were used to determine pore volume, pore size and the surface areas of the samples.

#### Zeta Potential

Electrokinetic measurements were obtained at 25°C in a PBS (pH 7.2) between each functionalization step using a Zetasizer Nano apparatus (Malvern Instruments, United Kingdom). The zeta potential was calculated: on the basis of the Schmolukowski model.

#### Fourier Transform Infrared (FT-IR) Analysis

Chemical analyses of MSNs, FITC-labeled MSNs and CADY-coated FITC-labeled MSNs were carried out using an FT-IR spectrophotometer (Model 100 series, PerkinElmer, Inc.). The Spectra were recorded at ambient temperature over a wave number range of 4,000–400 cm^–1^ at a 2 cm^–1^ resolution based on an average of 64 scans.

#### Ultraviolet Visible (UV-Vis) Spectroscopy

UV-Vis spectroscopic analysis of TP was measured with an ABTRONICS Model No. LT2900 spectrophotometer in the range of 200–400 nm.

### Statistical Analysis

The data are expressed as the mean ± SEM. All statistical analyses were performed using SPSS version 17.0. ANOVAs with *post hoc* LSD (SPSS, Chicago, IL, United States) were used to analyze data among groups, while Student’s *t*-tests were used for all statistical analyses. A *p*-value of < 0.05 was considered statistically significant.

## Results

### miR-30-5p Was Overexpressed in RA Clinical Tissues

**FIGURE 1:**
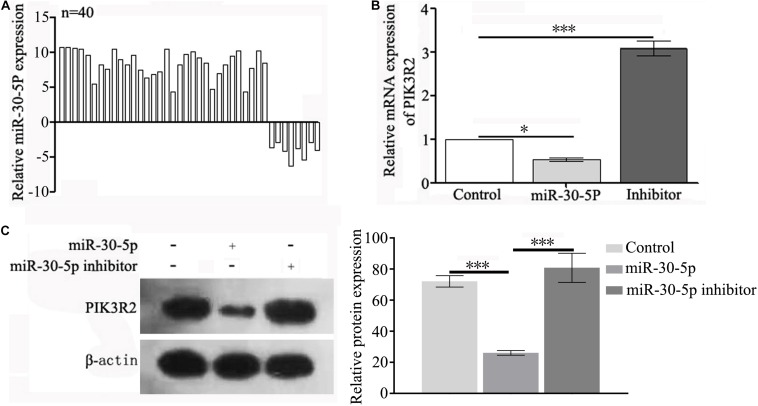
miR-30-5p is overexpressed in RA clinical tissues and regulates the expression of PIK3R2 in the studied cell lines. **(A)** Relative mRNA expression of miR-30-5p in samples from 40 patients (28 males and 12 females) undergoing surgery was tested by qPCR. **(B)** Relative mRNA expression of PIK3R2 in HFLS cells transfected with miR-30-5p or miR-30-5p inhibitor, as determined by qPCR. **(C)** Protein expression of PIK3R2 in HFLS cells transfected with miR-30-5p or miR-30-5p inhibitor, as determined by Western blotting. Experiments in **(B,C)** were repeated three independent times (^∗^*p* < 0.05, ^∗∗^*p* < 0.01, and ^∗∗∗^*p* < 0.001).

To analyze the expression levels of miRNAs in RA patients, we screened tissues from 40 RA patients and 15 healthy volunteers, and we identified that miR-30-5p was overexpressed in 32 RASFs (32/40, 80%) compared with expression in the corresponding adjacent tissues (Figure 1A). Using TargetScan Human 7.2, we found that PIK3R2 was a putative target of miR-30-5p. Then, in the HFLS cells transfected with miR-30-5p, both the mRNA and protein level of PIK3R2 were decreased significantly, while transfection with the miR-30-5p inhibitor resulted in a significant increase in PIK3R2, suggesting that miR-30-5p targeted PIK3R2 (Figures 1B,C and Supplementary Figure 1).

### Synthesis and Characterization of the MSNs and Surface Modifications

**FIGURE 2:**
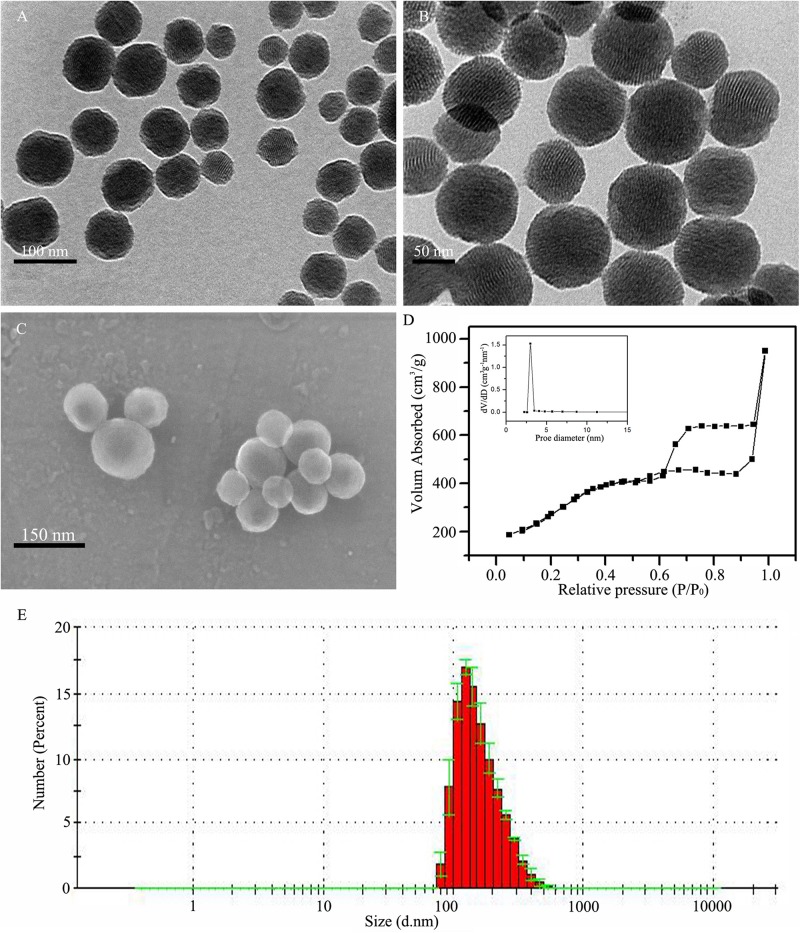
Synthesis and characterization of MSNs. **(A,B)** TEM image of MSNs at different magnifications. **(C)** SEM image of MSNs. **(D)** The mesoporous morphology and mesoporous distribution (inset) of MSNs were determined by the N_2_ adsorption-desorption isotherms. **(E)** Dynamic light scattering (DLS) determination of the particle diameter of MSNs.

Nanoparticles with stable structures are the basis of drug carrier construction. In this study, MSNs were constructed as previously described with some modifications (Biswas, 2017), and their structure was characterized. The results of the TEM (Figures 2A,B), SEM (Figure 2C), and DLS (Figure 2E) analyses showed that the particle sizes of the MSNs were 120–150 nm. The N_2_ adsorption-desorption isotherms showed that the mesoporous distribution of the MSNs was approximately 3 nm (Figure 2D).

### MSN Modifications and Drug Loading

**FIGURE 3:**
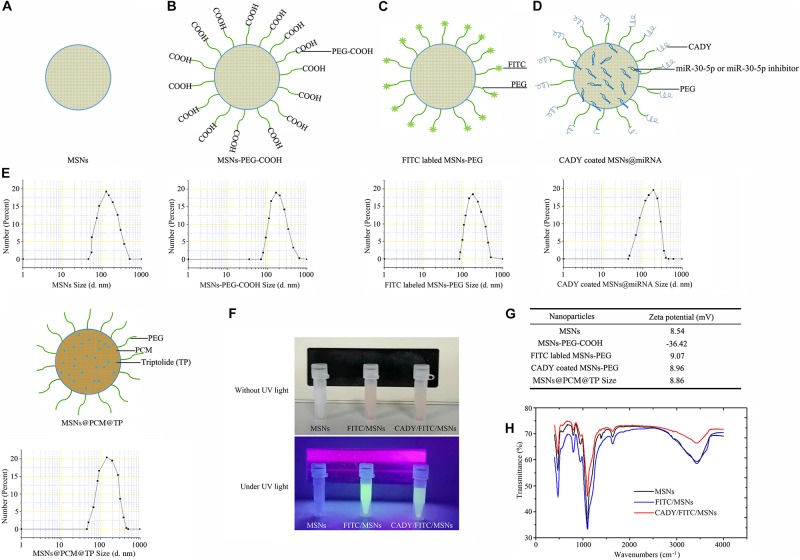
Schematic of the procedures: DLS, zeta potential and FT-IR spectra of different nanoparticles. **(A–E)** Patterns of and DLS results of MSNs, MSNs-PEG-COOH, FITC-labeled MSNs-PEG, CADY-coated MSNs@miRNA, and MSNs@PCM@TP. **(F)** FITC-labeled MSNs and CADY-coated FITC-labeled MSNs were irradiated by an ultraviolet lamp. **(G)** Zeta potential of the MSNs, MSNs-PEG-COOH, FITC-labeled MSNs-PEG, CADY coated MSNs and TP-loaded MSNs-PEG. **(H)** FT-IR spectra of MSNs, FITC-labeled MSNs and CADY-coated FITC- labeled MSNs.

Separate MSNs are easily aggregated in hydrophilic solvents, which affects MSN application. PEG-COOH modification on the surface of MSNs can resolve the nanoparticle aggregation issue. Similarly, polypeptides and fluorescent groups can be added to the surface of MSNs through carboxyl groups. In this study, we constructed MSNs@CADY for the smooth delivery of gene-targeting drugs into cells with FITC modification of the MSN surfaces to track the entry of nanodrug carriers into cells. Figures 3A–E shows the patterns and DLS test results for different nanoparticles (MSNs, MSNs-PEG-COOH, FITC-labeled MSNs-PEG, CADY-coated MSNs@miRNA, and MSNs@PCM@TP). Zeta potential was used to detect the surface modification effect of the nanoparticles. As shown in Figure 3F, FITC-labeled MSNs or CADY coated FITC-labeled MSNs emitted bright green fluorescence upon irradiation with an ultraviolet lamp, indicating that these modifications can be used to trace the nanodrug carriers. Compared with the zeta potential of the MSNs (8.54), the zeta potential of the MSNs@PEG-COOH, FITC-labeled MSNs and CADY-coated MSNs was 36.42, 9.07, and 8.96 fold, respectively (Figure 3G). The FT-IR spectra of the MSNs, FITC-labeled MSNs and CADY coated FITC-labeled MSNs are shown in Figure 3H and indicate that the surface of the MSNs was successfully modified by FITC and CADY.

### Cytotoxicity Induced by MSNs@PEG-COOH in 293, HFLS and HL-7702 Cells

**FIGURE 4:**
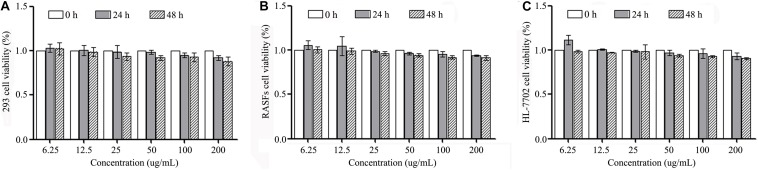
Cytotoxicity induced by MSNs@PEG-COOH in **(A)** 293 cells, **(B)** HFLS cells, and **(C)** HL-7702 cells after treatment for 24 and 48 h. Experiments were repeated three independent times.

The cytotoxicity induced by the MSNs-PEG-COOH in the 293, HFLS and HL-7702 cells was tested using an MTT cell activity detection assay. The results showed that MSNs-PEG-COOH had no cytotoxicity at concentrations of 6.25–200 μg/ml after 24 and 48 h of incubation (Figures 4A–C).

### Cellular Internalization of FITC-Labeled MSNs and FITC-Labeled MSNs@CADY

One of the key characteristics of nanodrug carriers is their ability to effectively deliver drugs into cells. Modification of membrane-penetrating peptides on the surface of drug carriers can effectively improve the efficiency of drug carriers against the cell membrane barrier. The secondary amphipathic peptide, CADY, has low cytotoxicity and no immunogenicity and has been verified to enhance the efficiency of drug uptake by cells (Rittner et al., 2002; Crombez et al., 2009; Konate et al., 2016).

**FIGURE 5:**
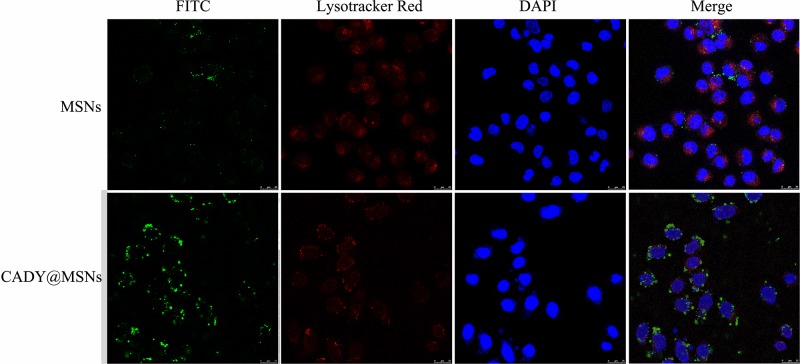
Internalization characteristics of FITC-labeled MSNs and FITC-labeled MSNs@CADY by HFLS cells treated for 12 h, as observed by confocal laser scanning microscopy. Cells were stained with DAPI and LysoTracker Red DND-99.

In this study, to better introduce nanodrug carriers into HFLS cells, CADY modified MSNs (MSNs@CADY) were constructed, and FITC was used as a tracer for the MSNs. A total of 100 μg/ml FITC-labeled MSNs and FITC-labeled MSNs@CADY were separately incubated with HFLS cells for 12 h. As shown in Figure 5, compared with the amount of MSNs, more MSNs@CADY (green fluorescence) was observed in and around the HFLS cells.

### miR-30-5p Inhibitor-Loaded MSNs@CADY Can Inhibit Proliferation and Promote the Apoptosis of RASFs by Targeting PIK3R2

**FIGURE 6:**
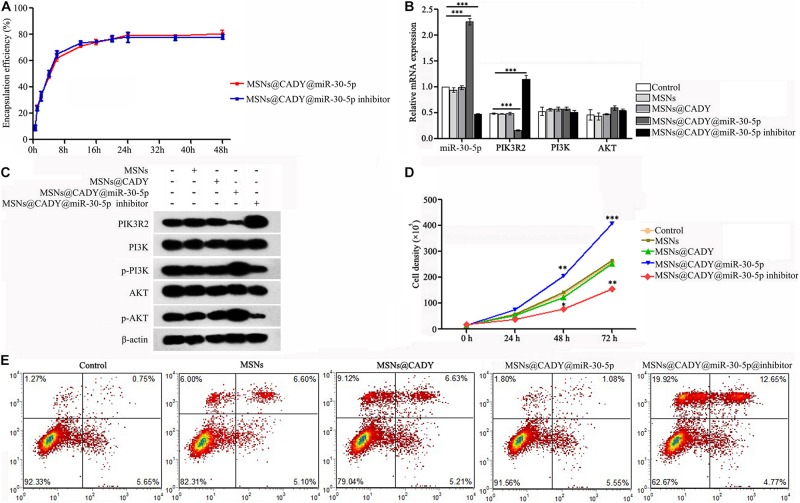
miR-30-5p inhibitor-loaded MSNs@CADY inhibited the proliferation and promoted the apoptosis of RASF cells by targeting PIK3R2. **(A)** The encapsulation efficiency of MSNs@CADY@miR-30-5p and MSNs@CADY@miR-30-5p inhibitor for miR-30-5p and miR-30-5p inhibitor was determined using semipermeable dialysis bags in PBS (pH 7.4) at 37°C. RASFs were treated with 100 μg/ml MSNs, MSNs@CADY, MSNs@CADY@miR-30-5p and MSNs@CADY@miR-30-5p inhibitor for 24 h. **(B)** Relative mRNA expression of miR-30-5p, PIK3R2, PI3K, and AKT as determined by qPCR. **(C)** Relative protein expression of PIK3R2, PI3K, p-PI3K, AKT, and p-AKT as determined by Western blot. **(D)** The cell number was determined after treatment with different nanodrug carriers for 0, 24, 48, and 72 h. The graph represents the cell growth curve for each group. **(E)** The RASF apoptosis level in each group was measured by Annexin V/PI staining. Experiments were repeated three independent times (^∗^*p* < 0.05, ^∗∗^*p* < 0.01, and ^∗∗∗^*p* < 0.001).

PI3K/AKT is a signaling pathway with high activity in SFs, and its abnormal activation can create an imbalance in SF proliferation and apoptosis. The miR-30-5p inhibitor loaded MSNs@CADY was constructed. According to the loading capacity results, 11.57 mg of miR-30-5p and 11.96 mg of miR-30-5p inhibitor were loaded in 1 mg of MSNs@CADY. The encapsulation efficiency of MSNs@CADY@miR-30-5p was similar to that of MSNs@CADY@miR-30-5p inhibitor, and both were able to stably release microRNA for at least 48 h (Figure 6A). RASFs were treated with 100 μg/ml MSNs, MSNs@CADY, MSNs@CADY@miR-30-5p or MSNs@CADY@miR-30-5p inhibitor for 24 h, and the expression of PIK3R2, PI3K, p-PI3K, AKT, and p-AKT was analyzed. As shown in Figures 6B,C and Supplementary Figure 2, the MSNs@CADY@miR-30-5p inhibitor upregulated the expression of PIK3R2 and downregulated the expression of p-PI3K and p-AKT. We also examined the proliferation and apoptosis of RASFs upon treatment them with nanodrug carriers and found that the MSNs@CADY@miR-30-5p inhibitor suppressed RASF proliferation (Figure 6D) and promoted RASF apoptosis (Figure 6E) in RASFs, suggesting that the MSNs@CADY@miR-30-5p inhibitor can suppress the growth of RASFs.

### MSNs@PCM@TP Under 808 nm Laser Downregulated Activation of the Immune System in the RA Rat Model

*Tripterygium wilfordii* has been used clinically as an immunosuppressive agent in recent years. TP, an effective component in *Tripterygium wilfordii*, is one of the most important anti-inflammatory and immunosuppressive reagents that has been demonstrated in clinical trials (Ziaei and Halaby, 2016). However, a large amount of evidence has shown that TP induces a certain degree of toxicity mainly in the heart, liver, bone marrow, chest, spleen, kidney, and reproductive system (Zhen et al., 1995; Shamon et al., 1997). Near infrared response nanodrug carriers have been used to improve many toxic drug formulations due to controlled release performance. Liu et al. (2005) constructed TP-PLA-NPs, which were found to enhance the antirheumatic inflammatory effect and decrease the toxicity of TP in the kidney, liver, and testis.

**FIGURE 7:**
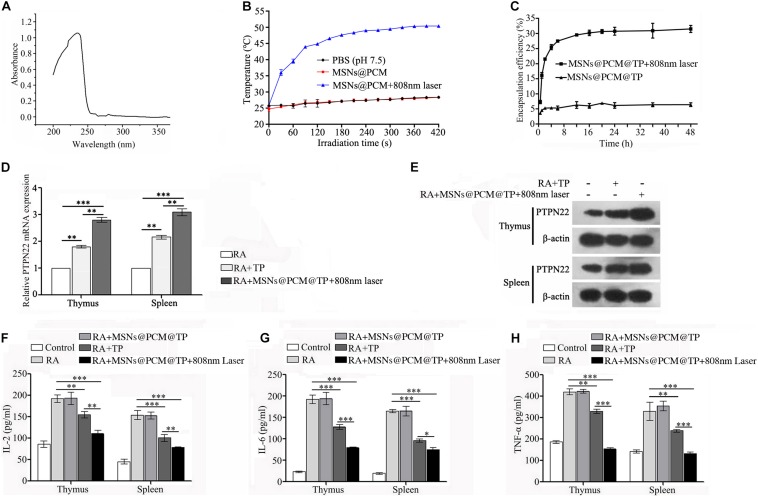
MSNs@PCM@TP downregulated the activation of the immune system in the RA rat model. **(A)** The maximum absorption peak of TP in PBS (pH 7.4) was found by full wavelength scanning. **(B)** The temperature changing of MSNs@PCM under 808 nm laser. **(C)** Encapsulation efficiency profiles of the MSNs@PCM@TP for TP were determined by use of semipermeable dialysis bags in PBS (pH 7.4) at 37°C. **(D,E)** mRNA and protein expression of PTPN22 in the thymus and spleen of RA rat treated with 100 μg/ml MSNs@PCM@TP under 808 nm laser irradiation for 1 week as detected by qPCR and Western blot assay, respectively. **(F–H)** Serum levels of IL-2, IL-6, and TNF-α in rats treated with 100 μg/ml MSNs@PCM@TP under 808 nm laser irradiation for 1 week, as determined by ELISA. Experiments were repeated three independent times (^∗^*p* < 0.05, ^∗∗^*p* < 0.01, and ^∗∗∗^*p* < 0.001).

Here, we loaded TP together with PCM onto the MSNs and specifically tested the capacity of 230 nm MSNs to load TP (Figure 7A). The results showed that 1 mg of MSNs could load as much as 45.27 μg of TP. The temperature change of on MSNs@PCM under 808 nm laser was shown in Figure 7B, according to the results, under 808 nm laser, the temperature of MSNs@PCM increases rapidly, after 120s of irradiation, the temperature can rise to about 45°C, and finally to about 50°C. We then tested the encapsulation and release efficiency of the MSNs@PCM@TP in PBS at pH 7.4 with or without 808 nm laser. As shown in Figure 7C, under 808 nm laser, the cumulative drug release rate reached approximately 30% after 12 h, followed by a sustained slow release for a long period of time. However, without 808 nm laser irradiation, almost no TP is released from the MSNs@PCM@TP.

PTPN22 is a negative regulator of immune activation and plays an important role in RA (Clarke et al., 2017, 2018; Carmona and Martin, 2018). To detect the effect of the MSNs@PCM@TP + 808 nm laser on the expression of PTPN22 in RA rats, we compared the mRNA and protein levels of PTPN22 in the thymus and spleen of rats treated with 100 μg/ml of MSNs@PCM@TP + 808 nm laser for 1 week with those of the RA and RA + TP groups. Both the mRNA and protein levels of PTPN22 were found to increase significantly in the MSNs@PCM@TP + 808 nm laser group (Figures 7D,E and Supplementary Figure 3). The protein levels of IL-2, IL-6, and TNF-α in rat serum were also measured. According to the results, MSNs@PCM@TP + 808 nm laser significantly decreased the protein levels of IL-2, IL-6, and TNF-α compared with the levels in the free TP group (Figures 7F–H).

### The Combination of MSNs@CADY@miR-30-5p Inhibitor and MSNs@PCM@TP Has Great Potential for RA Treatment

Drugs are administered in different ways according to their mechanism of action and mode of absorption (Qian et al., 2019). Synergistic treatments with different drugs and different administration modes are widely used in clinical settings, and the therapeutic effects of combinations are often better than those of each individual drug alone. Therefore, we implemented a combination of I.C. MSNs@CADY@miR-30-5p inhibitor and I.P. MSNs@PCM@TP + 808 nm laser to treat RA rats.

**FIGURE 8:**
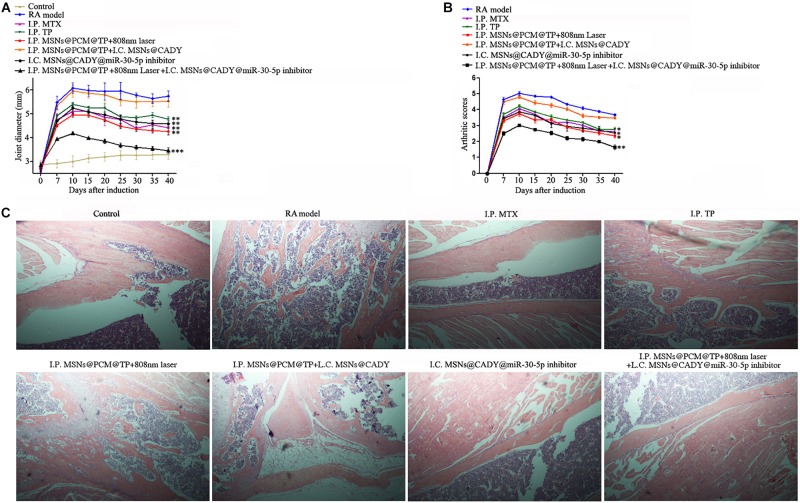
Therapeutic potential of the combination of MSNs@CADY@miR-30-5p inhibitor and MSNs@PCM@TP + 808 nm laser in the treatment of RA. Rats treated with I.C. MSNs@CADY + I.P. MSNs@PCM@TP, I.C. MSNs@CADY@miR-30-5p inhibitor, I.P. MSNs@PCM@TP + 808 nm laser and I.C. MSNs@CADY@miR-30-5p inhibitor + I.P. MSNs@PCM@TP + 808 nm laser were examined for joint diameter, arthritis score, and joint histological damage. **(A)** Joint diameter of each group (*n* = 6). **(B)** Arthritis score of each group (*n* = 6). **(C)** Histological images of rat ankle joints.

Firstly, the biocompatibility of MSNs, MSNs@CADY and MSNs@PCM were tested in mice. According to the results, a final concentration of 500 μg/ml MSNs, MSNs@CADY and MSNs@PCM had caused no significant toxic reaction in the experimental group, and there was no significant difference in the weight change between the experimental group and the PBS control group (Supplementary Figure 4). For the animal experiments, we used the two most common drugs for RA, TP and MTX, as positive controls. Longitudinal measurements indicated that the joint diameter and arthritis scores of the rats treated with I.C. MSNs@CADY@miR-30-5p inhibitor alone and of the rats treated with I.P. MSNs@PCM@TP + 808 nm laser were significantly lower than those of the I.P. MTX and TP control group rats. Importantly, the effect of I.C. MSNs@CADY@miR-30-5p inhibitor + I.P. MSNs@PCM@TP + 808 nm laser was significantly greater than the effect of either I.C. MSNs@CADY@miR-30-5p inhibitor or I.P. MSNs@PCM@TP + 808 nm laser alone (*p* < 0.001, Figures 8A,B). F Results of histological examination showed severe articular cartilage destruction and inflammatory cell infiltration in the bone tissue in the RA groups. In contrast, a nearly healthy bone structure and articular cartilage surface were observed in the I.C. MSNs@CADY@miR-30-5p inhibitor + I.P. MSNs@PCM@TP + 808 nm laser group, and the therapeutic effect in the I.C. MSNs@CADY@miR-30-5p inhibitor + I.P. MSNs@PCM@TP + 808 nm laser group was also greater than that in the I.P. MTX and I.P. TP group (Figure 8C). These results indicate that the combination of MSNs@CADY@miR-30-5p inhibitor and MSNs@PCM@TP + 808 nm laser could be a potential treatment option for RA.

## Discussion

As a chronic autoimmune disease, rheumatoid arthritis mainly affects joint tissue and promotes osteopenia around the joint (Tanaka and Ohira, 2018). In this study, TP plus miR-30-5p inhibitor delivered via modified MSNs was evaluated for its therapeutic efficacy in RA.

Currently, RA treatment focuses on suppressing the overactive immune system and maintaining the balance between the proliferation and apoptosis of SFs. Given the rapid development of RA treatment drugs, most patients can control their RA symptoms. However, due to the reduced distribution of the RA drugs in diseased joints after administration, the curative effect is not ideal, and adverse reactions are severe. Furthermore, these drugs generally have poor bioavailability and must be administered frequently and in high doses (Xiao et al., 2009). Therefore, the adverse reactions of normal tissues to RA drugs limit the widespread clinical use of these drugs. Targeted therapy can deliver drugs to the lesion site, tissue or specific cells, providing a way to resolve the problems described above. As a targeted drug delivery system, nanopreparations have been widely studied and applied. At the cellular level, the PI3K/AKT signaling pathway phosphorylates and regulates many proteins related to cell metabolism, apoptosis, proliferation and differentiation, and inhibits cell apoptosis and promotes tumor growth. PIK3R2 is a negative regulator of the PI3K/AKT signaling pathway (Stanczyk et al., 2008; Song et al., 2016). PIK3R2 maintaining the balance between proliferation and apoptosis of SFs (Fan et al., 2018). Our results showed miR-30-5p overexpression and PIK3R2 downregulation in 32 of 40 clinical cases of RA. The expression of PIK3R2 decreased significantly in SFs transfected with miR-30-5p, which indicates that the inhibition of miR-30-5p can be used to maintain the balance between SF proliferation and apoptosis by increasing PIK3R2.

To improve the efficiency of miR-30-5p inhibitor in the RASFs, cell membrane penetrating peptide CADY-modified MSNs with good biocompatibility and release characteristics were constructed. The results show that the cell entry efficiency of MSNs@CADY was significantly greater than that of MSNs. MSNs@CADY@miR-30-5p inhibitor effectively inhibited proliferation and promote apoptosis of the RASFs by targeting PIK3R2 to regulate the PI3K/AKT signaling pathway.

Tripterygium Wilfordii Hook F is a traditional Chinese herbal medicine used to treat RA. It has good efficacy and is also cost effective. TP is the main active ingredient in TWHF (Li et al., 2014). TP can inhibit the secretion of inflammatory cytokines and chemical factors by inhibiting the transcription activities of purine-box and NF-κB in epidermal cells, monocytes, human peripheral blood lymphocytes and T cells. Previous studies have shown that TP can also interfere with the expression of proinflammatory factors in LPS-stimulated mouse macrophages and can significantly inhibit their induction *in vivo* (Moore et al., 2001; Min et al., 2004). However, the use of TP is restricted because of the toxic side effects it induces in the heart, liver, bone marrow, chest, spleen, kidney, and reproductive system. Many studies have verified that loading TP into nanoparticles with good release properties is useful in reducing its cytotoxicity by reducing the local concentration of TP and prolonging the time of drug action (Mei et al., 2003; Liu et al., 2008). To ameliorate the side effects induced by TP, we loaded TP into near infrared response photothermal controlled-release MSNs, to control the release time of TP. According to the results, under 808 nm laser, the cumulative drug release rate reached approximately 30% after 12 h followed by a sustained slow release for a long period of time. However, without 808 nm laser irradiation, almost no TP was released from MSNs@PCM@TP. In subsequent animal experiments, MSNs@PCM@TP + 808 nm laser increased the mRNA and protein levels of PTPN22 in the thymus and spleen in the RA rats, while it decreased the protein levels of IL-2, IL-6 and TNF-α in RA rat serum, thereby achieving the objective of inhibiting the over activated immune system. Finally, I.C. MSNs@CADY@miR-30-5p inhibitor plus I.P. MSNs@PCM@TP + 808 nm laser were used in combination to treat RA rats, and the combination therapy significantly improved the therapeutic effect on RA by affecting different pathways of the overactive immune system and the imbalance of SFs.

In summary, this study demonstrated that the efficient delivery of triptolide plus miR-30-5p inhibitor using modified MSNs has therapeutic efficacy in RA treatment. The results of the pharmacodynamic study showed that the combination of MSNs@CADY@miR-30-5p inhibitor and MSNs@PCM@TP + 808 nm laser relieved joint swelling to some extent and inhibited articular cartilage destruction in the synovium. These findings suggest that the effective release of the traditional Chinese medicine TP and the newly identified miR-30-5p inhibitor through the use of modified MSNs could lead to the development of innovative therapeutic approaches for RA.

## Data Availability Statement

Publicly available datasets were analyzed in this study. This data can be found here: http://www.targetscan.org/vert_72/.

## Ethics Statement

The studies involving human participants were reviewed and approved by The Ethical Board of China-Japan Union Hospital of Jilin University. The patients/participants provided their written informed consent to participate in this study. The animal study was reviewed and approved by The Institutional Animal Care and Use Committee of Northeastern University.

## Author Contributions

XaZ performed the majority of the experiments, analyzed the data, wrote and edited the manuscript. YY analyzed the data and helped with revising the manuscript. XnZ collected and processed clinical samples. XW, TW, BB, NZ, and YZ advised on parts of the study. BW directed the study, analyzed and approved all of the data, wrote and edited the manuscript. All authors reviewed the manuscript.

## Conflict of Interest

The authors declare that the research was conducted in the absence of any commercial or financial relationships that could be construed as a potential conflict of interest.

## References

[B1] Altman R. Asch E. Bloch D. Bole G. Borenstein D. Brandt K. (1986). Development of criteria for the classification and reporting of osteoarthritis. Classification of osteoarthritis of the knee. Diagnostic and therapeutic criteria committee of the American Rheumatism Association. *Arthritis Rheum.* 29 1039–1049. 10.1002/art.1780290816 3741515

[B2] Arnett F. C. Edworthy S. M. Bloch D. A. McShane D. J. Fries J. F. Cooper N. S. (1988). The American Rheumatism Association 1987 revised criteria for the classification of rheumatoid arthritis. *Arthritis Rheum.* 31 315–324. 10.1002/art.1780310302 3358796

[B3] Biswas N. (2017). Modified mesoporous silica nanoparticles for enhancing oral bioavailability and antihypertensive activity of poorly water soluble valsartan. *Eur. J. Pharm. Sci.* 99 152–160. 10.1016/j.ejps.2016.12.015 27993684

[B4] Cai H. Liang Z. Huang W. Wen L. Chen G. (2017). Engineering PLGA nano-based systems through understanding the influence of nanoparticle properties and cell-penetrating peptides for cochlear drug delivery. *Int. J. Pharm.* 532 55–65. 10.1016/j.ijpharm.2017.08.084 28870763

[B5] Carmona F. D. Martin J. (2018). The potential of PTPN22 as a therapeutic target for rheumatoid arthritis. *Expert Opin. Ther. Targets* 22 879–891. 10.1080/14728222.2018.1526924 30251905

[B6] Choi S. W. Zhang Y. Xia Y. (2010). A temperature-sensitive drug release system based on phase-change materials. *Angew. Chem. Int. Ed. Engl.* 49 7904–7908. 10.1002/anie.201004057 20839209 PMC3008559

[B7] Clarke F. Jordan C. K. Gutierrez-Martinez E. Bibby J. A. Sanchez-Blanco C. Cornish G. H. (2017). Protein tyrosine phosphatase PTPN22 is dispensable for dendritic cell antigen processing and promotion of T-cell activation by dendritic cells. *PLoS One* 12:e0186625. 10.1371/journal.pone.0186625 29040339 PMC5645108

[B8] Clarke F. Purvis H. A. Sanchez-Blanco C. Gutierrez-Martinez E. Cornish G. H. Zamoyska R. (2018). The protein tyrosine phosphatase PTPN22 negatively regulates presentation of immune complex derived antigens. *Sci. Rep.* 8:12692. 10.1038/s41598-018-31179-x 30139951 PMC6107551

[B9] Collison J. (2016). Rheumatoid arthritis: tipping the balance towards resolution. *Nat. Rev. Rheumatol.* 12:622 10.1038/nrrheum.2016.159 27652507

[B10] Crombez L. Aldrian-Herrada G. Konate K. Nguyen Q. N. McMaster G. K. Brasseur R. (2009). A new potent secondary amphipathic cell-penetrating peptide for siRNA delivery into mammalian cells. *Mol. Ther.* 17 95–103. 10.1038/mt.2008.215 18957965 PMC2834975

[B11] Dowaidar M. Abdelhamid H. N. Hallbrink M. Freimann K. Kurrikoff K. Zou X. (2017). Magnetic nanoparticle assisted self-assembly of cell penetrating peptides-oligonucleotides complexes for gene delivery. *Sci. Rep.* 7:9159. 10.1038/s41598-017-09803-z 28831162 PMC5567346

[B12] Fan D. Guo Q. Shen J. Zheng K. Lu C. Zhang G. (2018). The effect of triptolide in rheumatoid arthritis: from basic research towards clinical translation. *Int. J. Mol. Sci.* 19:376. 10.3390/ijms19020376 29373547 PMC5855598

[B13] Fan D. He X. Bian Y. Guo Q. Zheng K. Zhao Y. (2016). Triptolide modulates TREM-1 signal pathway to inhibit the inflammatory response in rheumatoid arthritis. *Int. J. Mol. Sci.* 17:498 10.3390/ijms17040498 PMC4848954 27049384

[B14] Gao J. Zhou X. L. Kong R. N. Ji L. M. He L. L. Zhao D. B. (2016). microRNA-126 targeting PIK3R2 promotes rheumatoid arthritis synovial fibro-blasts proliferation and resistance to apoptosis by regulating PI3K/AKT pathway. *Exp. Mol. Pathol.* 100 192–198. 10.1016/j.yexmp.2015.12.015 26723864

[B15] Jeong E. J. Choi M. Lee J. Rhim T. Lee K. Y. (2015). The spacer arm length in cell-penetrating peptides influences chitosan/siRNA nanoparticle delivery for pulmonary inflammation treatment. *Nanoscale* 7 20095–20104. 10.1039/c5nr06903c 26568525

[B16] Kalaria D. R. Sharma G. Beniwal V. Ravi Kumar M. N. (2009). Design of biodegradable nanoparticles for oral delivery of doxorubicin: in vivo pharmacokinetics and toxicity studies in rats. *Pharm. Res.* 26 492–501. 10.1007/s11095-008-9763-4 18998202

[B17] Konate K. Lindberg M. F. Vaissiere A. Jourdan C. Aldrian G. Margeat E. (2016). Optimisation of vectorisation property: a comparative study for a secondary amphipathic peptide. *Int. J. Pharm.* 509 71–84. 10.1016/j.ijpharm.2016.05.030 27224007

[B18] Kong X. Zhang Y. Liu C. Guo W. Li X. Su X. (2013). Anti-angiogenic effect of triptolide in rheumatoid arthritis by targeting angiogenic cascade. *PLoS One* 8:e77513. 10.1371/journal.pone.0077513 24204851 PMC3810371

[B19] Larsen A. Dale K. Eek M. (1977). Radiographic evaluation of rheumatoid arthritis and related conditions by standard reference films. *Acta Radiol. Diagn. (Stockh)* 18 481–491. 10.1177/028418517701800415 920239

[B20] Le Goff B. Soltner E. Charrier C. Maugars Y. Redini F. Heymann D. (2009). A combination of methotrexate and zoledronic acid prevents bone erosions and systemic bone mass loss in collagen induced arthritis. *Arthritis Res. Ther.* 11:R185. 10.1186/ar2877 20003278 PMC3003529

[B21] Li X. J. Jiang Z. Z. Zhang L. Y. (2014). Triptolide: progress on research in pharmacodynamics and toxicology. *J. Ethnopharmacol.* 155 67–79. 10.1016/j.jep.2014.06.006 24933225

[B22] Liu M. Dong J. Yang Y. Yang X. Xu H. (2005). Anti-inflammatory effects of triptolide loaded poly(D,L-lactic acid) nanoparticles on adjuvant-induced arthritis in rats. *J. Ethnopharmacol.* 97 219–225. 10.1016/j.jep.2004.10.031 15707756

[B23] Liu M. X. Dong J. Yang Y. J. Yang X. L. Xu H. B. (2008). Preliminary research on abating rat testicle toxicity due to triptolide after oral polymer nanoparticle delivery. *Drug Discov. Ther.* 2 188–193. 22504571

[B24] Manchanda R. Fernandez-Fernandez A. Nagesetti A. McGoron A. J. (2010). Preparation and characterization of a polymeric (PLGA) nanoparticulate drug delivery system with simultaneous incorporation of chemotherapeutic and thermo-optical agents. *Colloids Surf. B Biointerfaces* 75 260–267. 10.1016/j.colsurfb.2009.08.043 19775872

[B25] Mei Z. Chen H. Weng T. Yang Y. Yang X. (2003). Solid lipid nanoparticle and microemulsion for topical delivery of triptolide. *Eur. J. Pharm. Biopharm.* 56 189–196. 10.1016/s0939-6411(03)00067-5 12957632

[B26] Min S. Y. Hwang S. Y. Park K. S. Lee J. S. Lee K. E. Kim K. W. (2004). Induction of IL-10-producing CD4+CD25+ T cells in animal model of collagen-induced arthritis by oral administration of type II collagen. *Arthritis Res. Ther.* 6 R213–R219. 10.1186/ar1169 15142267 PMC416445

[B27] Moon G. D. Choi S. W. Cai X. Li W. Cho E. C. Jeong U. (2011). A new theranostic system based on gold nanocages and phase-change materials with unique features for photoacoustic imaging and controlled release. *J. Am. Chem. Soc.* 133 4762–4765. 10.1021/ja200894u 21401092 PMC3073071

[B28] Moore K. W. de Waal Malefyt R. Coffman R. L. O’Garra A. (2001). Interleukin-10 and the interleukin-10 receptor. *Annu. Rev. Immunol.* 19 683–765. 10.1146/annurev.immunol.19.1.683 11244051

[B29] Mullen M. B. Saag K. G. (2015). Evaluating and mitigating fracture risk in established rheumatoid arthritis. *Best Pract. Res. Clin. Rheum.* 29 614–627. 10.1016/j.berh.2015.09.005 26697770

[B30] Onozaki K. (2009). Etiological and biological aspects of cigarette smoking in rheumatoid arthritis. *Inflamm. Allergy Drug Targets* 8 364–368. 10.2174/1871528110908050364 20025584

[B31] Qian K. Zhang L. Shi K. (2019). Triptolide prevents osteoarthritis via inhibiting hsa-miR-20b. *Inflammopharmacology* 27 109–119. 10.1007/s10787-018-0509-6 29974310

[B32] Qu Y. Zhang Y. P. Wu J. Jie L. G. Deng J. X. Zhao D. B. (2019). Downregulated microRNA-135a ameliorates rheumatoid arthritis by inactivation of the phosphatidylinositol 3-kinase/AKT signaling pathway via phosphatidylinositol 3-kinase regulatory subunit 2. *J. Cell. Physiol.* 234 17663–17676. 10.1002/jcp.28390 30912120

[B33] Rittner K. Benavente A. Bompard-Sorlet A. Heitz F. Divita G. Brasseur R. (2002). New basic membrane-destabilizing peptides for plasmid-based gene delivery in vitro and in vivo. *Mol. Ther.* 5 104–114. 10.1006/mthe.2002.0523 11829517

[B34] Rosenholm J. M. Meinander A. Peuhu E. Niemi R. Eriksson J. E. Sahlgren C. (2009). Targeting of porous hybrid silica nanoparticles to cancer cells. *ACS Nano* 3 197–206. 10.1021/nn800781r 19206267

[B35] Rosenholm J. M. Peuhu E. Bate-Eya L. T. Eriksson J. E. Sahlgren C. Linden M. (2010). Cancer-cell-specific induction of apoptosis using mesoporous silica nanoparticles as drug-delivery vectors. *Small (Weinheim an der Bergstrasse, Germany)* 6 1234–1241. 10.1002/smll.200902355 20486218

[B36] Saxena V. Sadoqi M. Shao J. (2004). Indocyanine green-loaded biodegradable nanoparticles: preparation, physicochemical characterization and in vitro release. *Int. J. Pharm.* 278 293–301. 10.1016/j.ijpharm.2004.03.032 15196634

[B37] Sha L. Wang D. Mao Y. Shi W. Gao T. Zhao Q. (2018). Hydrophobic interaction mediated coating of pluronics on mesoporous silica nanoparticle with stimuli responsiveness for cancer therapy. *Nanotechnology* 29:345101. 10.1088/1361-6528/aac6b1 29786605

[B38] Shamon L. A. Pezzuto J. M. Graves J. M. Mehta R. R. Wangcharoentrakul S. Sangsuwan R. (1997). Evaluation of the mutagenic, cytotoxic, and antitumor potential of triptolide, a highly oxygenated diterpene isolated from *Tripterygium wilfordii*. *Cancer Lett.* 112 113–117. 10.1016/s0304-3835(96)04554-5 9029176

[B39] Song L. Li D. Gu Y. Wen Z. M. Jie J. Zhao D. (2016). MicroRNA-126 targeting PIK3R2 inhibits NSCLC A549 cell proliferation, migration, and invasion by regulation of PTEN/PI3K/AKT pathway. *Clin. Lung Cancer* 17 e65–e75. 10.1016/j.cllc.2016.03.012 27236384

[B40] Stanczyk J. Pedrioli D. M. Brentano F. Sanchez-Pernaute O. Kolling C. Gay R. E. (2008). Altered expression of MicroRNA in synovial fibroblasts and synovial tissue in rheumatoid arthritis. *Arthritis Rheum.* 58 1001–1009. 10.1002/art.23386 18383392

[B41] Tanaka Y. Ohira T. (2018). Mechanisms and therapeutic targets for bone damage in rheumatoid arthritis, in particular the RANK-RANKL system. *Curr. Opin. Pharmacol.* 40 110–119. 10.1016/j.coph.2018.03.006 29702364

[B42] Tang Q. Chang S. Tian Z. Sun J. Hao L. Wang Z. (2017). Efficacy of indocyanine green-mediated sonodynamic therapy on rheumatoid arthritis fibroblast-like synoviocytes. *Ultrasound Med. Biol.* 43 2690–2698. 10.1016/j.ultrasmedbio.2017.06.030 28779958

[B43] Wang T. Jiang H. Wan L. Zhao Q. Jiang T. Wang B. (2015). Potential application of functional porous TiO2 nanoparticles in light-controlled drug release and targeted drug delivery. *Acta Biomater.* 13 354–363. 10.1016/j.actbio.2014.11.010 25462846

[B44] Wang Y. Kou J. Zhang H. Wang C. Li H. Ren Y. (2018). The renin-angiotensin system in the synovium promotes periarticular osteopenia in a rat model of collagen-induced arthritis. *Int. Immunopharmacol.* 65 550–558. 10.1016/j.intimp.2018.11.001 30412852

[B45] Xiao C. Zhou J. He Y. Jia H. Zhao L. Zhao N. (2009). Effects of triptolide from radix *Tripterygium wilfordii* (Leigongteng) on cartilage cytokines and transcription factor NF-kappaB: a study on induced arthritis in rats. *Chin. Med.* 4:13. 10.1186/1749-8546-4-13 19570240 PMC2709898

[B46] Xue X. Gong L. Qi X. Wu Y. Xing G. Yao J. (2011). Knockout of hepatic P450 reductase aggravates triptolide-induced toxicity. *Toxicol. Lett.* 205 47–54. 10.1016/j.toxlet.2011.05.003 21596114

[B47] Zhang J. Liu L. Mu X. Jiang Z. Zhang L. (2012). Effect of triptolide on estradiol release from cultured rat granulosa cells. *Endocr. J.* 59 473–481. 10.1507/endocrj.ej11-0407 22447140

[B48] Zhen Q. S. Ye X. Wei Z. J. (1995). Recent progress in research on *Tripterygium*: a male antifertility plant. *Contraception* 51 121–129. 10.1016/0010-7824(94)00018-r 7750290

[B49] Ziaei S. Halaby R. (2016). Immunosuppressive, anti-inflammatory and anti-cancer properties of triptolide: a mini review. *Avicenna J. Phytomed.* 6 149–164. 27222828 PMC4877967

[B50] Zwerina J. Redlich K. Schett G. Smolen J. S. (2005). Pathogenesis of rheumatoid arthritis: targeting cytokines. *Ann. N. Y. Acad. Sci.* 1051 716–729. 10.1196/annals.1361.116 16127012

