# Supplementary material for: Efficient Delivery of Triptolide Plus a miR-30-5p Inhibitor Through the Use of Near Infrared Laser Responsive or CADY Modified MSNs for Efficacy in Rheumatoid Arthritis Therapeutics
Source: Front Bioeng Biotechnol. 2020 Mar 17;8:170. doi: 10.3389/fbioe.2020.00170 (PMC7092621; doi:10.3389/fbioe.2020.00170)
Supplement: Supplementary file 1 [file Data_Sheet_1.docx]

**Efficient delivery of triptolide plus a miR-30-5p inhibitor through the use of near infrared laser responsive or CADY modified MSNs for efficacy in rheumatoid arthritis therapeutics**

Xiaonan Zhang^a^, Xin Zhang^b^, Xipeng Wang^a^, Tao Wang^a^, Bin Bai^a^, Na Zhang^a^, Yanjiao Zhao^a^, Yang Yu*^a^, Bing Wang*^a^

^a^Institute of Biochemistry and Molecular Biology, College of Life and Health Sciences, Northeastern University, Shenyang, 110819, P. R. China

^b^Department of Rheumatology and Immunology, China-Japan Union Hospital of Jilin University, Changchun, 130033, P.R. China

**Supplementary materials**

**
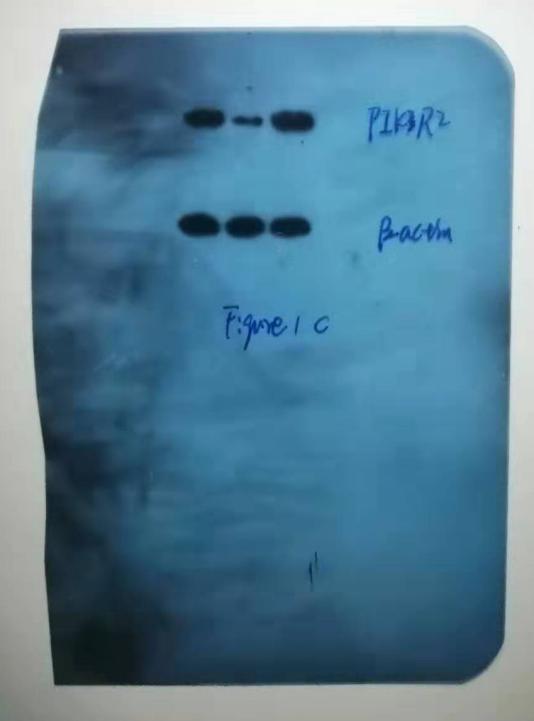
**

Supplementary figure 1 The original data of figure 1C


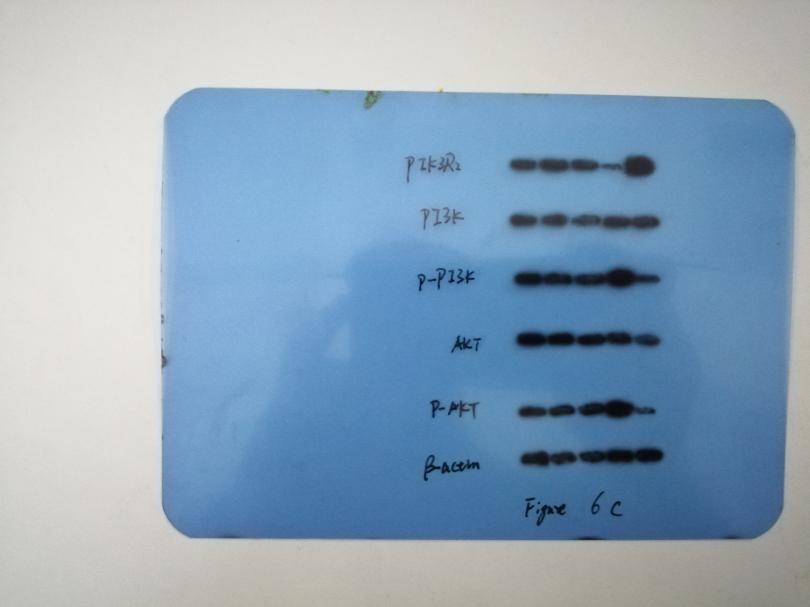


Supplementary figure 2 The original data of figure 6C


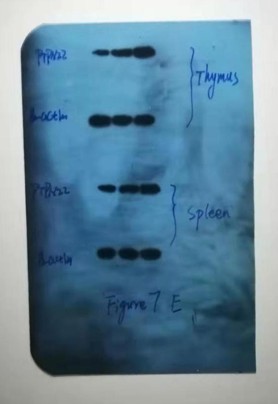


Supplementary figure 3 The original data of figure 7E

**The biocompatibility of MSNs, MSNs@CADY and MSNs@PCM in mice**

48 mice were randomly divided into 4 groups (PBS control group, 500 ug/mL MSNs, 500 ug/mL MSNs@CADY, 500 ug/mL MSNs@PCM), 6 male and 6 female in each group. All mice were deprived of food for 12h and then injected with nanoparticles at a dose of 100mg/kg, while the control group was given the same volume of PBS. The toxicity of the mice was observed for 7 days. The time in which symptoms appear, worsen, reduce or die, and the number of animals with these reactions were recorded. Subsequently, the mortality of animals was calculated. The results showed that a final concentration of 500 ug/mL MSNs, MSNs@CADY and MSNs@PCM had caused no significant toxic reaction in the experimental group, and there was no significant difference in the weight change between the experimental group and the PBS control group (Supplementary figure 4).


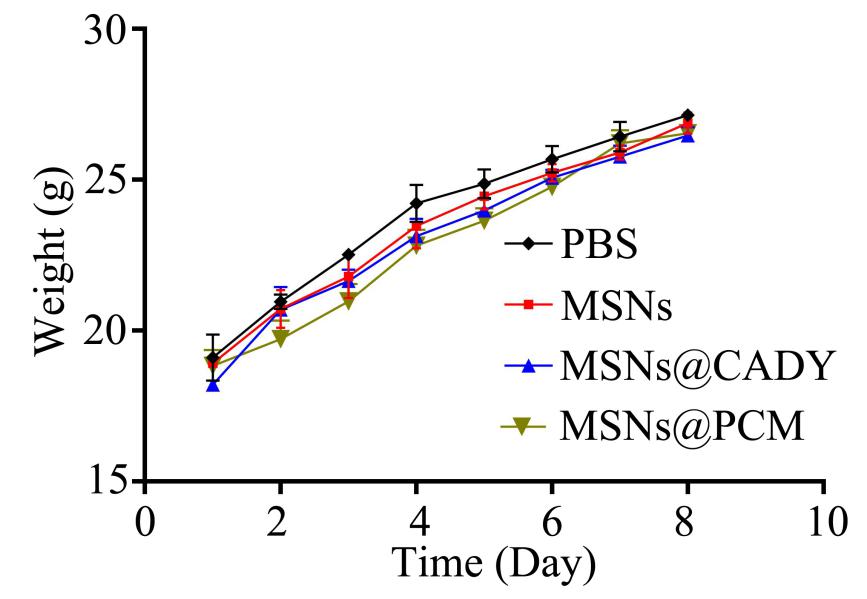


Supplementary figure 4 Effects of MSNs, MSNs@CADY and MSNs@PCM on body weight of mice.

| Genes | Primers |
| --- | --- |
| miR-30a-5p | F 5’-GGGTGTAAACATCCTCGAC-3’  R 5’-CAGTGCGTGTCGTGGAGT-3’ |
| U6 | F 5’-CTCGCTTCGGCAGCACA-3’;  R 5’-AACGCTTCACGAATTTGCGT-3’ |
| PTPN22 | F 5’-CCGAATTCCGATGGACCAAAGAGAAATTCTGC-3’  R 5’-CCGAATTCGGCATGGACCAAAGAGAAATTCTGC-3’ |
| PIK3R2 | F 5'-GCACCACGAGGAACGCACTT-3'  R 5'-CGTCCACTACCACGGAGCAG-3' |
| AKT | F 5'-CGGATCCTGACCAAAAACC-3'  R 5'-TCATGATGTCCGTGGTCAAT-3' |
| GAPDH | F 5'-GCACCGAGTTCGCTGCATTAT-3'  R 5'-GCCTATTATTATACGTCCCATG-3' |

**Supplementary table 1. Primers used in the article**
